# Quality of multiple-choice questions in medical internship qualification examination determined by item response theory at Debre Tabor University, Ethiopia

**DOI:** 10.1186/s12909-022-03687-y

**Published:** 2022-08-22

**Authors:** Lalem Menber Belay, Tegbar Yigzaw Sendekie, Fantu Abebe Eyowas

**Affiliations:** 1 Jhpiego, Bahir Dar, Ethiopia; 2 Jhpiego, Addis Ababa, Ethiopia

**Keywords:** Medical students, Qualification exam, Multiple-choice question, Psychometrics

## Abstract

**Background:**

Assessment of cognitive competence is a major element of the internship qualification exam in undergraduate medical education in Ethiopia. Assessing the quality of exam items can help to improve the validity of assessments and assure stakeholders about the accuracy of the go/no decision to the internship. However, we know little about the quality of exam items utilized to ascertain fitness to join the medical internship. Therefore, this study aimed to analyze the quality of multiple-choice questions (MCQs) of the qualification exam administered to final-year medical students at Debre Tabor University (DTU), Ethiopia.

**Methods:**

A psychometric study was conducted to assess the qualities of 120 randomly selected MCQs and 407 distractors. Item characteristics were estimated using the item response theory (IRT) model. T-test, one-way ANOVA, and chi-square tests were run to analyze the univariate association between factors. Pearson’s correlation test was done to determine the predictive validity of the qualification examination.

**Result:**

Overall, 16, 51, and 33% of the items had high, moderate, and low distractor efficiency, respectively. About two-thirds (65.8%) of the items had two or more functioning distractors and 42.5% exhibited a desirable difficulty index. However, 77.8% of items administered in the qualification examination had a negative or poor discrimination index. Four and five option items didn’t show significant differences in psychometric qualities. The qualification exam showed a positive predictive value of success in the national licensing examination (Pearson’s correlation coefficient = 0.5).

**Conclusions:**

The psychometric properties of the medical qualification exam were inadequate for making valid decisions. Five option MCQs were not better than four options in terms of psychometric qualities. The qualification examination had a positive predictive validity of future performance. High-stakes examination items must be properly created and reviewed before being administered.

**Supplementary Information:**

The online version contains supplementary material available at 10.1186/s12909-022-03687-y.

## Background

The vision for universal health coverage by 2030 is believed to be realized by putting quality care as a priority issue along with access, coverage, and affordability [1]. Ensuring the quality of pre-service education is a prerequisite for quality of healthcare, however, the growing need to train more health workers, coupled with rapid expansion in medical knowledge, presents a serious challenge to the quality of health professions’ education [2]. The Ethiopian Government has emphasized the need to produce competent health care workers in its five-year health sector transformation plan (HSTP) [3]. In response to the nation’s call for quality education, Debre Tabor University (DTU) developed an integrated, problem-based, and competency-based medical curriculum [4].

In a competency-based curriculum, greater emphasis is given to the quality of assessment [5–7]. Competence assessment ensures mastery of essential competencies [8, 9], and has a powerful effect on the quality of education and healthcare [9]. The effectiveness of content and methods of learning depend to a major extent on how students think they will be assessed [5, 9–11]. Valid and reliable assessment can elicit true knowledge and abilities, discriminate between high and low performers, reinforce students’ learning and inspire them to be competent, and can be stored, retrieved, and used again in the future [10, 12–14].

A variety of assessment methods are used in health professions education, each method having its intrinsic strengths and weaknesses [9, 15]. The single best answer MCQs (type-A MCQs) are the most flexible and dominant assessment formats in health professions’ education [8, 9, 15–20]. High-quality MCQs have a stem, lead-in, and options set, are context-rich, and measure higher-order cognitive skills, ethics, and professionalism [9, 18, 21–23].

Ensuring the quality of exam items can be done before, during, and after test administration [24]. Posttest psychometric analyses help to understand, monitor and improve the quality of MCQs [8, 10, 22, 25, 26]. Assessment tools should have sufficient psychometric values to ensure the validity of decisions [9, 27]. Item parameters are determined using either the Classical Test Theory (CTT) or the Item Response Theory (IRT); the CTT is cohort-dependent, and student performance is item-dependent [25, 28], however, the IRT solves the limitations of CTT and provides a much more detailed analysis to provide diagnostic feedback to objective test items [26]. In IRT, the probability of a student answering an item correctly is a function of the item's difficulty and the student's ability [29].

Evidence shows the validity of assessment results is affected by the content tested, quality of test items, qualification of item writers, number of test items, presence of item writing flaws, and psychometric characteristics of items [5, 9–11, 14, 20, 30, 31]. Item difficulty, measured by the percentage of examinees that correctly answered the item, runs from 0 to 1; easy items have a higher difficulty index [32]. Most studies classify item difficulty as too easy (≥ 0.8), moderately easy (0.7–0.8), desirable (0.3–0.7), and difficult (< 0.3) [22, 33–37]. A study at Lourdes College, Sylvania, Ohio showed that 63, 14, 21, and 2% of MCQs in the final nursing exam were too easy, moderately easy, desirable, and too difficult, respectively [22]. Another study at the department of pathology, K. S. Hegde Medical Academy, India showed that 85, 5, and 10% of MCQs administered to medical students were desirable, easy, and difficult, respectively [37].

The item discrimination index, which measures the item's ability to distinguish high performers from low performers, runs from -1 to + 1 [38] with a desired value ≥ 0.30 [39]. A study in India showed that 60, 10, 15, and 15% of MCQs had excellent (*DI* > 0.4), good (*DI* = 0.3–0.39), acceptable (*DI* = 0.2–0.29), and poor (*DI* < 0–0.19) discriminating abilities respectively [37]. Items with DI ≥ 0.2 are acceptable whereas negatively discriminating items need to be reviewed or removed [40]. Item discrimination and difficulty indexes have been shown to be positively correlated [37]; moderately difficult MCQs have better discriminating ability [37, 40], and difficult items tend to have negative discrimination [6].

Distracters are alternative answers to the correct answer in a multiple-choice question that are designed to attract less knowledgeable students. Creating functioning distractors is a difficult task in MCQ construction [9, 20–22]. Published studies reported wide variation in functional distractors (31.6 to 95%) [33, 35, 37, 41]. A study by Sajjad, M., et al. found that 20% of the MCQs had low distractor efficiency [41]. Another study by Fozzard, N., et al. showed that 32% of MCQs had only three effective distractors, 7% did not have any effective distractor and there was no difference in item performance between four and five options MCQs [28]. MCQs with many non-functioning distractors (NFDs) are easier and have lower discrimination ability [40]; correction of NFDs improved the discriminatory power of MCQs [20]. Likewise, flawed items, testing low cognitive function, and low distractor efficiency have a negative impact on the item difficulty and discrimination indexes [20, 42]. Peer-review of MCQs improved the psychometric characteristics of the items [43], and short-term faculty development programs increased item's ability to assess higher cognitive functions, decreased item writing flaws, and increased distractors efficiency and mean score of students [31, 40, 44, 45].

The reliability of individual items and an entire test is measured by point bi-serial coefficient [14, 39] and Kuder-Richardson reliability index (KR-20), respectively [39]. High stake exams, end-of-course or end-of-year exams, and classroom-type exams require reliability of greater or equal to 0.90, 0.80, and 0.70, respectively [13].

Undergraduate medical education at Debre Tabor University is organized into two years of pre-clerkship, three years of clerkship, and one year of internship. The undergraduate medical qualification exam was administered to final-year medical students before transitioning to the internship. A variety of assessment methods (written examination, objective structured clinical examination (OSCE), and oral examinations) are used in the qualification examination. However, we knew little about the psychometric qualities and the predictive validity of the qualification exam. Therefore, the study aimed to address the following research questions:

- Was there a defined procedure used during the exam development process to assure quality?
- Are the psychometric qualities of the MCQ items acceptable for high-stakes exams (difficulty index, discrimination index, reliability, and distracters efficiency)?
- Can the outcome of the qualification exam be used to predict future performance?

## Methods

### Study design and setting

A psychometric study was conducted to assess the quality of MCQs of the qualification examination administered to fifth-year medical students at Debre Tabor University, Ethiopia. 

### Study participants

The study analyzed the quality of MCQs administered to 44 medical students who sat for the qualification examination in December 2019. We randomly selected 120 out of 396 MCQs using a systematic random sampling technique. The sample size was calculated using the single population proportion formula with the assumptions of a 95% confidence interval, 50% proportion of MCQs with acceptable discrimination index, a 5% margin of error, and applying the finite population correction. Since no previous study findings were available, we used a 50% proportion of MCQs with acceptable DI to achieve the maximum sample size. The only criteria to include items was their availability. We used the scores of 42 of the 44 medical students who completed their internship program and took the licensure examination to determine the correlation between the qualification and licensure examination. Data on the item development process was collected from key informants.

### Data collection

The qualification exam papers were collected from the four major clinical departments of DTU. Data on the academic background and demographic characteristics of students were collected from the registrar, and students’ performance in the national licensure examination was obtained from the Health Professionals Competency Assessment and Licensure Directorate (HPCALD) of the Ministry of Health. Data related to the exam development process was collected by interviewing department heads of internal medicine, surgery, pediatrics and gynecology/obstetrics, and the HSEDC (Health Science Education Development Center) coordinator. The qualification exam covered surgery, pediatrics, gynecology & obstetrics, internal medicine, emergency medicine, radiology, ophthalmology, ENT, dermatology, and social and population health (SPH) courses. However, exam items on internal medicine, emergency medicine, and dermatology were unavailable.

Items were sorted into five groups based on their discrimination value: excellent (≥ 0.4), good (0.3–0.39), acceptable (0.2–0.29), poor (0–0.19), and negative (< 0). Similarly, based on difficulty index, items were categorized into four groups: hard (0–29%), desirable (30–70%), moderate easy (71–79%), and easy (> = 80%). We calculated the number of functional distractors (i.e., options selected by ≥ 5% of examinees) per item (#FDs/item) [14]. Since 73 of the items were 4 option and 47 items were 5-option, we calculated the percentages of distractor efficiency separately (Table 1): For 4-option items, 3FDs/item (100% DE), 2FDs/item (66.6%DE), 1FD/item (33.3%DE), and 0FD/item (0%DE) and for 5-option items, 4FDs/item (100%DE), 3FDs/item (75% DE), 2FDs/item (50%DE), 1FD/item (25%DE), and 0FD/item (0%DE).

**Table 1:** Distractor Efficiency of Multiple-Choice Question Items [41]

Number of FDs		Distractor efficiency
Four options	Five options	
3	4	High (100%)
2	2–3	Moderate (50–75%)
0 -1	0–1	Low (< 50%)

Before beginning the data collection, the principal investigator explained the purpose of the study and answered questions, presented a letter of ethical approval from Jimma University to the school dean, department heads, and the Director of the Health Professionals Competency Assessment, and Licensure Directorate at Ministry of Health.

### Data analysis

The item difficulty index, discrimination index, reliability, and distractor functionality were determined using the item response theory (IRT) model [26]. The performance of students was demonstrated by the item characteristics curve (ICC) and test characteristics curve (TCC). Percentage of MCQs having excellent, good, acceptable, poor, and negative discrimination index; difficult, desirable, moderately easy, and easy difficulty levels; and high, moderate, and low DE were computed. Graphs and tables were used to present the result.

A univariate analysis was performed using a t-test, chi-square, one-way ANOVA, and Pearson’s correlation test after checking the normality of the data. The academic performance of male and female students (Table 2), as well as the mean number of functioning distracters, difficulty index, and discrimination index in four and five-option MCQs (Table 3) were compared using a t-test. The association between categorical variables (difficulty index, discrimination index, and distractor efficiency) and four versus five-option MCQs (Table 3) and difficulty index versus discrimination index was determined using a chi-square test (Table 4). One-way ANOVA was used to compare the mean number of functioning distractors in the categories of discrimination and difficulty indexes (Table 5). The relationship between the qualification and the licensure exams was determined using Pearson's correlation coefficient (Fig. 5).

The jMetrick version 4.1.1 software was used to determine psychometric qualities and to create item and test characteristics curves. The univariate analyses were carried out using STATA IC version 12 software. The statistical significance level was set at a *p*-value < 0.05.

## Results

### Profile of students

The study analyzed the performance of 42 medical students, 19 (45%) male and 23 (55%) female, who took both the qualification and licensure examinations. The mean pre-internship cumulative GPA was 3.17 and qualification and licensure exam scores were 66.1 and 67.4%, respectively. There was no meaningful difference in the performance of male and female students (Table 2).

**Table 2:** Mean qualification and licensure exam scores, and pre-internship cumulative GPA of students at DTU, 2021

**Variable**	**Male(***n*** = 19)**	**Female(*n* = 23)**	**Total (*N* = 42)**	***p***
Licensure exam score, mean (SD)	66.3 (6.7)	68.4 [6.5)	67.4 (6.6)	0.308
Qualification exam score, mean (SD)	67.1(5.5)	65.3(6.2)	66.1(5.9)	0.329
Pre-internship CGPA, mean (SD)	3.17 (0.3)	3.18 (0.3)	3.17 (0.27)	0.899

### Item development process

The health science education development center (HSEDC) coordinated the qualification exam development process. Item developers received three days’ capacity-building training on exam blueprint and item development before they constructed the items. Items were prepared individually within two weeks of the training. But there was no standardized institutional guideline on item development for high-stake exams. The exam committee reviewed the items for homogeneity of the distractors, presence of technical item flaws that add irrelevant difficulty or cueing the correct answer, etc. but there wasn’t editorial, sensitivity, internal, and external content reviews. Also, field testing and psychometric analysis were not done. The exam included items from Surgery, Pediatrics, Gynecology & Obstetrics, Internal Medicine, Emergency Medicine, Radiology, Ophthalmology, ENT (Ear, Nose, and Throat), Dermatology, and Social and Population Health (SPH) courses. The four major clinical departments organized, administered, and marked the exam. Items developed to assess public health and so-called minor clinical attachments were embedded with major clinical courses.

### Psychometrics quality of multiple-choice questions

The study determined the psychometric qualities of 120 MCQs and 407 distractors (Additional file 1). The mean item difficulty level was 58% (95%CI: 53—63%). Of the reviewed items, 51(42.5%) MCQs had a desirable difficulty (0.3–0.7), and of which only 12(10%) had an acceptable discrimination index (DI ≥ 0.2). There was no significant difference in the item difficulty index between four and five option MCQs. Overall, 54(46.2%) and 37(31.6%) MCQs had poor and negative discrimination indexes, respectively. The mean item discrimination index was 0.08, and only 22% of MCQs were reusable (DI ≥ 0.2) Table 3).

Moreover, 19(15.8%), 61(50.8%), and 40(33.3%) of the items had high, moderate, and low distractor efficiency. Four options items had high distractor efficiency compared to five option items (Table 3). On average, items contained 1.8 functioning distractors and 216 (53.1%) distractors were functional (selected by ≥ 5% of the examinees). Eighteen (24.7%) of the four option items and only a single five option item had a 100% distractor efficiency (100%DE). Most 80(66.7%) of the items had at least two functioning distractors while 18(15%) MCQs didn’t have any functioning distractor (0%DE). Forty-nine (67.1%) and 31(65.9%) of four and five option items, respectively, had at least two functioning distractors per item (Table 3).

**Table 3:** Psychometric quality of MCQs utilized in undergraduate medical qualification exam, DTU, 2021

Item characteristics		Four option (*n* = 73)	Five option (*n* = 47)	Total (*N* = 120)	*p*
Difficulty level, n(%)	Difficult	10 (13.7)	11 (23.4)	22(18.3)	0.705
	Desirable	34 (46.6)	17 (36.2)	51(42.5)	
	Moderately easy	12 (16.4)	7 (14.9)	18(15.0)	
	Easy	17 (23.3)	12 (25.5)	29(24.2)	
Item discrimination, n(%)	Negative	23 (32.9)	14 (29.8)	37(31.6)	0.567
	Poor	34 (48.6)	20 (42.6)	54(46.2)	
	Acceptable	7 (10.0)	9 (19.2)	16(13.7)	
	Good	6 (8.6)	4 (8.5)	10(8.6)	
Number of functioning distractors per item	High	18(24.7%)	1(2.1%)	19 (15.8%)	0.666
	Moderate	31 (42.5%)	30(63.8%)	61 (50.8%)	
	Low	24 (32.9%)	16(34.0%)	40 (33.3%)	
Difficulty, mean (95%CI)		0.59 (0.53,0.63)	0.57(0.49,0.65)	0.58(0.53,0.63)	0.543
Discrimination, mean (95%CI))		0.07(0.03,0.11)	0.09(0.04,0.14)	0.08(0.05,0.11)	0.739
Total # distractors		219	188	407	
Total # FDs		129 (58.9)	87 (46.3)	216(53.1%)	
FDs/item, mean (95%CI)		1.8(1.5, 2.0)	1.9(1.5, 2.2)	1.8(1.61,1.99)	0.829

**Fig. 1:**
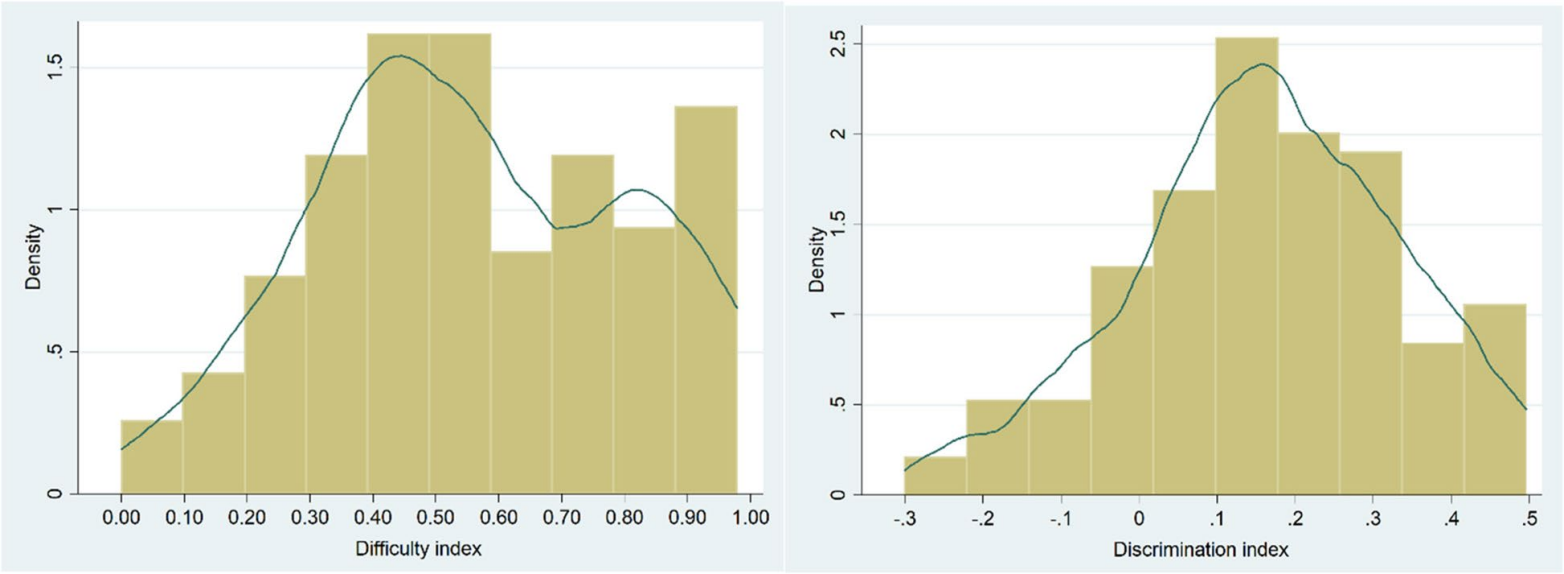
Density plot of item difficulty and discrimination indexes

The majority of the items (64%) had moderate to easy difficulty indexes with poor discrimination power (Additional file 2). The density plots also depicted that most of the items are moderately difficult and easy (Fig. 1). More than two-fifths, 51(42.5%), of the items had a desirable level of difficulty (0.3–0.7), of which only 12(10%) had an acceptable discrimination index (DI ≥ 0.2) (Table 4).

**Table 4:** Relationship between difficulty index and discrimination index of MCQs in the undergraduate medical qualification exam at DTU, 2021

**Difficulty index**	**Discrimination index**		Total	*p*-value
	Poor (Less than 0.2)	Acceptable (≥ 0.2)		
Difficult (less than 0.3)	16	4	20	0.945
Desirable (0.3–0.7)	39	12	51	
Easy (> 0.7)	36	10	19	
Total	91	26	117	

Difficult items contained a higher number of functioning distractors compared to easy items (*p*-value < 0.0001). But, we did not observe a statistically significant association between the discrimination index and the mean number of FDs/item (*p*-value = 0.3887) (Table 5).

**Table 5:** Mean number of functioning distractors of MCQs in the undergraduate medical qualification exam at DTU, 2021

Item characteristics	Category	Number of items	Mean # FDs	Std.dev	*p*-value
Difficulty index	Hard (0–0.29)	21	2.33	0.80	< 0.0001
	Desirable (0.3–0.7)	51	2.35	0.70	
	Moderate easy (0.71–0.79)	19	1.68	0.67	
	Easy (≥ 0.80)	29	0.52	0.69	
Discrimination index	Negative (< 0)	37	2.02	1.04	0.3887
	Poor (0–0.19)	54	1.72	0.98	
	Acceptable (0.2–0.29)	16	2.0	1.10	
	Good (0.3–0.39)	10	1.6	0.63	

### Item characteristics curves

**Fig. 2:**
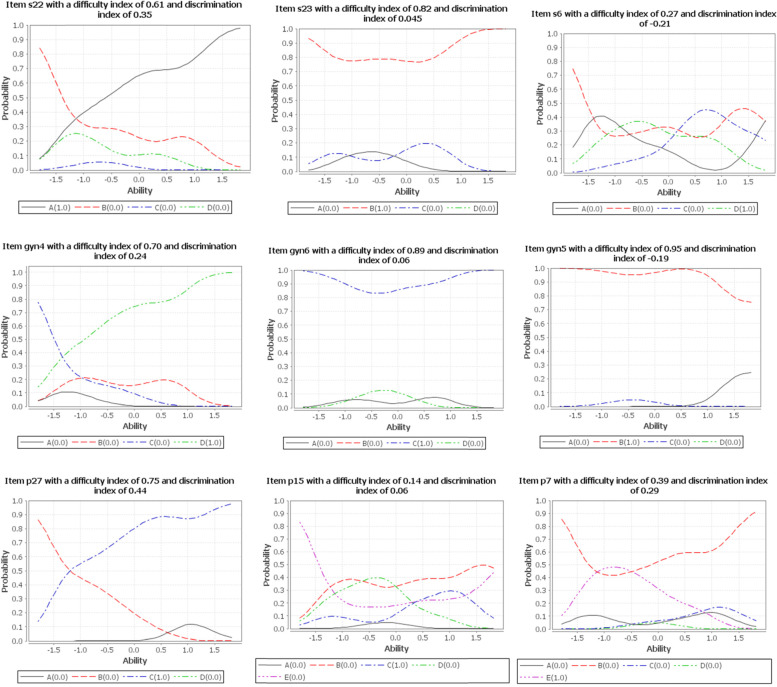
Item characteristics curves of sampled MCQ items, DTU, 2021. (A, B, C, D, E represent the options in the MCQ items and the key is indicated as (1,0))

A detailed analysis of the individual items through item characteristics curves (ICC) showed that moderately difficult items tended to have a positive discrimination index while difficult and easy items had a negative discrimination index (Fig. 2). Students’ ability was estimated using a two-parameter logistic regression model (2PL).

### Test characteristics curve

**Fig. 3:**
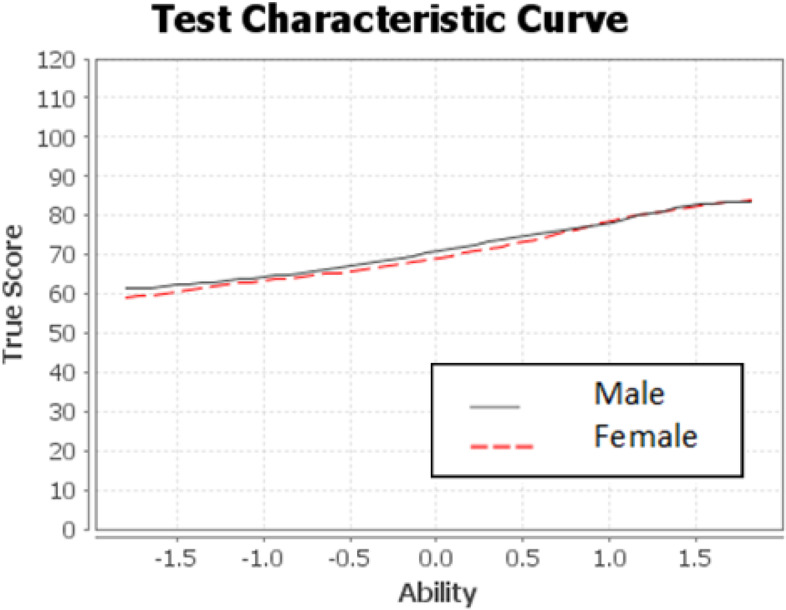
Test characteristics curve (TCC) of the qualification exam administered to the undergraduate medical students at DTU, 2021

The test characteristics curve revealed that as students' competence grew, so did their true score on the qualification examination (Fig. 3). The slope of the test characteristics curve (TCC) indicates how the true score is affected by students' abilities. The weak slope demonstrated that the qualification exam fails to distinguish between high and low performers (Fig. 3). The TCC also revealed that there was no significant difference in male and female students' performance.

### Correlation analysis

**Fig. 4:**
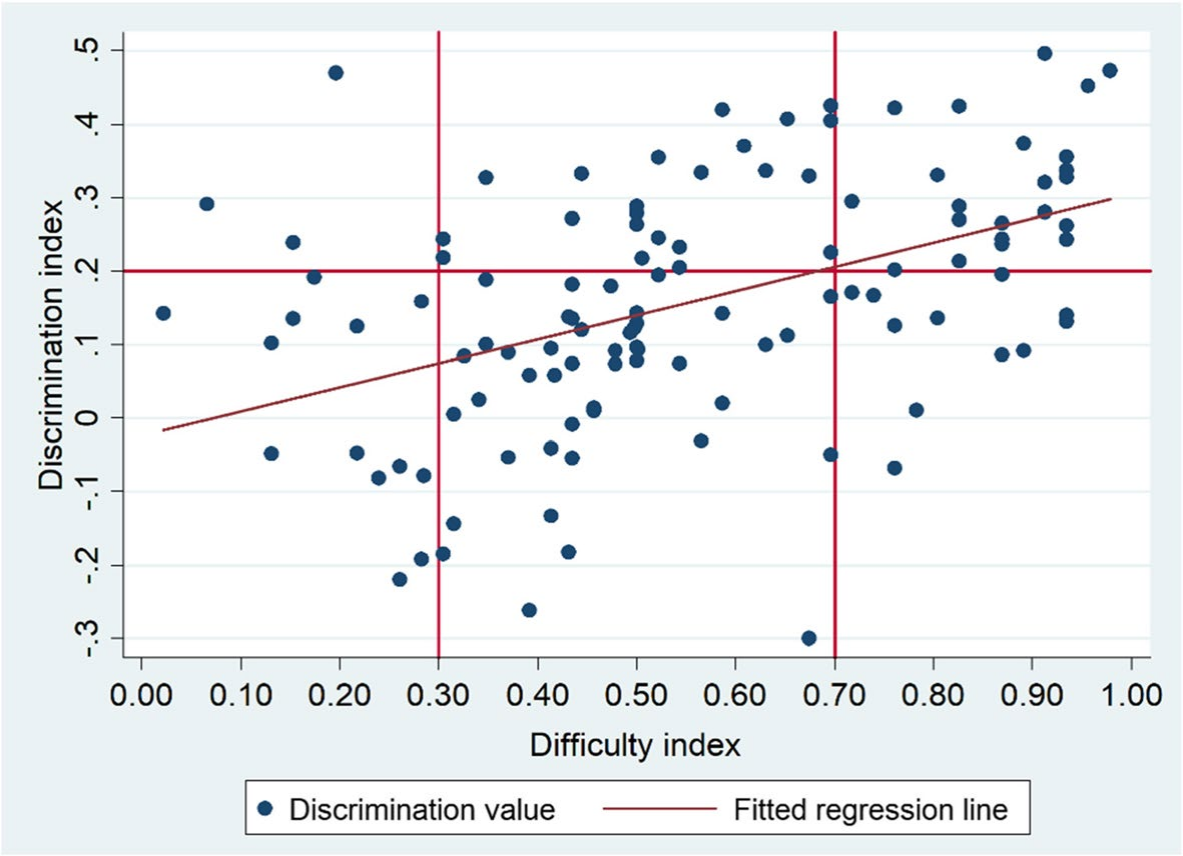
Correlation between item difficulty and discrimination indexes of the undergraduate medical qualification exam at DTU 2021

The Pearson’s correlation test showed a weak positive correlation between item difficulty and discrimination indexes (*r* = 0.1, *p* = 0.267). The graph also depicted that easy items had a better discrimination index compared to difficult items (Fig. 4).

**Fig. 5:**
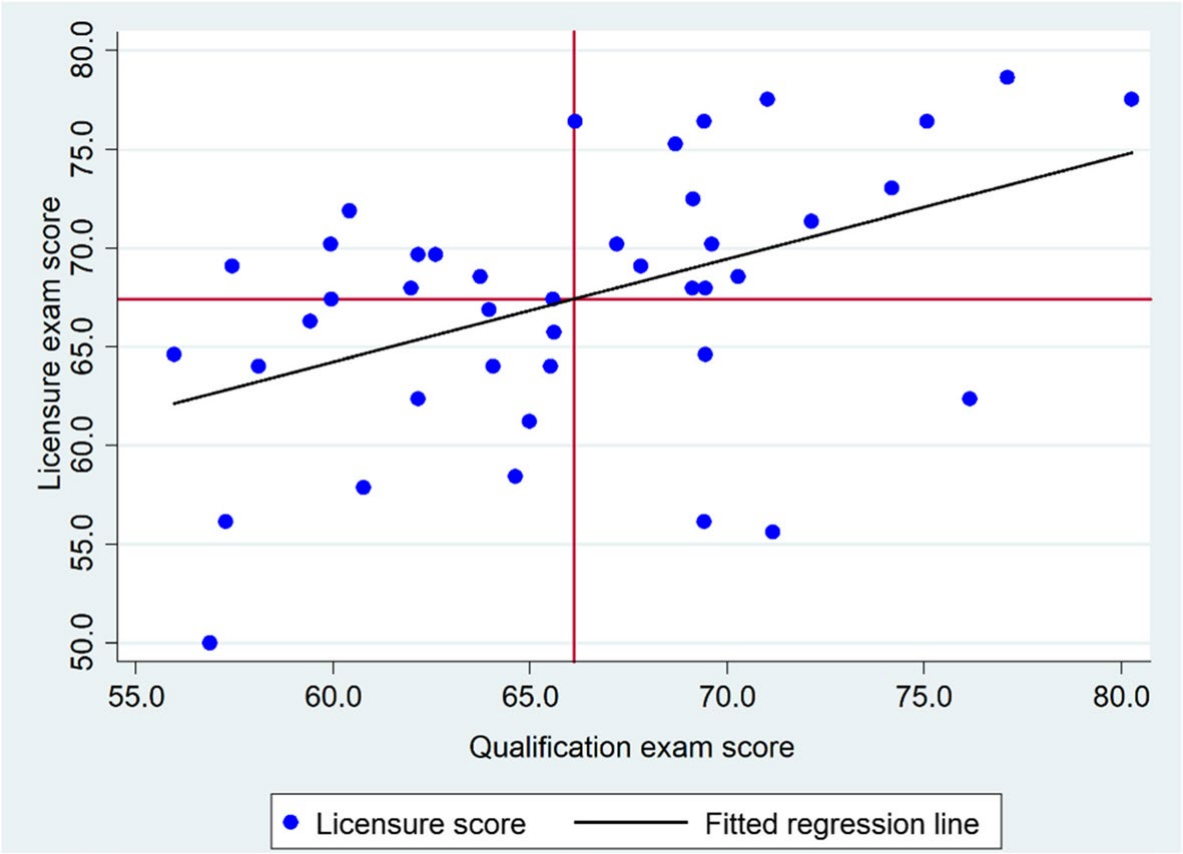
Correlation between the qualification exam and licensure exam results of 42 medical students at DTU, 2021

Similarly, a positive correlation was observed between the qualification and licensure exam scores (Pearson’s correlation coefficient, *r* = 0.5, *p* = 0.0018) (Fig. 5). Students who performed well in the qualification exam were more likely to succeed in the licensure examination.

## Discussion

The purpose of this study was to assess the quality of multiple-choice questions used in the medical internship qualification examination given to DTU students in December 2019. The qualification exam is a high-stake in-school assessment to ensure fitness to join medical internship. Ensuring the validity and reliability of the qualification exam is critical to protecting the public from incompetent medical interns.

Our findings demonstrated gaps in the quality of the qualification exam administered to fifth-year undergraduate medical students at DTU. There were major gaps in the discrimination index of items. Assessment tools should have sufficient psychometric values to ensure validity of decisions [9, 27]. Nonetheless, most of the items in this study had either poor or negative discrimination indexes (Table 5). In competency-based education, greater emphasis is given to the quality of student assessment because valid and reliable assessment drives the learning activities and ensures competence [5, 9]. While the difficulty index, reliability of items, and functionality of distractors were encouraging, the mean item discrimination was poor, making it difficult to be confident in the validity of the decisions. In line with the psychometric parameters, the test characteristics curve (TCC) showed a weak slope, which indicated that the exam was poor to distinguish between high and low-performing students. The TCC depicts the relationship between students' ability and their true scores (Fig. 3). The steepness of the curve reveals how well the exam differentiates between high and low-ability students; the steeper the curve, the stronger the exam in discriminating examinees. The item characteristics curve also depicted that easy and difficult items discriminate poorly, whereas moderately difficult items discriminate well.

Items having a discrimination index of 0.2 and above are acceptable for reuse [40]. The proportion of reusable items in our study was 22.3%, which is much lower than numbers reported in other settings, 54.7% in Malaysia [46], 60% in Qatar [47], and 85% in India [37]. The possible reasons for the poor discrimination index could be due to items that are ambiguously worded, miskeyed, or flawed that could benefit test-wise students. Studies showed that the removal of item writing flaws (IWFs) improves the discrimination power of the item [20]. In the current study, items were peer-reviewed for technical flaws, but no editorial, internal or external content review was done by experts before administration. This reinforces the need to evaluate the performance of items before using them in high-stake exams. In line with other study findings, difficult items tended to have a negative discrimination index in the present study [6].

Item writers aim to construct a higher proportion of moderately difficult items. The mean item difficulty index in this study was determined to be 58%, (95%CI: 54–63%) which is in the desirable range (0.3–0.7) [22, 34, 39]. Literatures showed a wide-ranging proportion (21–85%) of moderately difficult [22, 33, 36, 37, 48] and (2–19%) difficult items [22, 37, 47]. Our study showed that 42.5 and 15.8% of MCQs were moderately difficult and difficult, respectively. This shows a relatively fair proportion of moderately difficult and difficult items in the current study.

The most difficult task in writing high-quality MCQs is creating effective distractors. The distractor efficiency in our study was 53%, which is higher than the 31.6% [41] found in Pakistan but much lower than 95% [37], 85.3% [33], and 76.5% [35] reported in India, Pakistan, and Pakistan, respectively. The current study showed that 24.7% of four options items had a 100% distractor efficiency which was comparable with previous study results, 15%-30% [31, 48]. However, only a single five options item had a 100% distractor efficiency compared to 19% in the previous studies [28]. This shows that the present study had limitations in creating four effective distractors. Creating a functioning distractor is challenging for item writers when the number of options increases [19].

It is widely believed that increasing the number of distractors improves the quality of MCQs by decreasing the chance of guessing [19] and reducing the number of options make the item easier by increasing the probability of guessing [46]. However, our study showed no significant difference between four and five option items in terms of item difficulty, discrimination index, and functionality of distractors that corroborates findings from previously published studies [28]. In the present study, 67% of items contained at least two functioning distractors which was higher compared to 46.6% reported in Qatar [47]. Published studies showed that 7–20% of MCQs had no functioning distractors [28, 47, 49, 50] which was comparable to 15% in the current study.

The internal consistency of items (Cronbach alpha) was determined to be 0.91 which implies the items were measuring the same thing. This was in line with the recommended reliability for high-stake exams [14].

One way of evaluating the exam item quality is assessing the closeness of scores obtained on the reference (better quality) instrument of the same competency [14]. We hypothesized that items in the national licensure examination are of better quality because of the involvement of experts from different institutions in the item development and rigorous processes. Looking at the scatter plot in Fig. 3, the positive correlation between the qualification and licensure exams might provide a shred of evidence for the quality of MCQs used in the qualification exam. However, we would like the reader to note that this interpretation is made without assessing the quality of items used in the licensure examination.

### Strength and limitations of the study

To our knowledge, this is the first study in Ethiopia to report the psychometric qualities of a high-stake examination administered to medical students. However, the study has the following limitations. First, the findings of the study are based on data from a single exam in a single institution. Second, though we planned to analyze exam items from all courses, internal medicine, emergency medicine, and dermatology items were unavailable and not included. Therefore, the findings of the study may lack generalizability, and hence we invite other researchers to replicate the study in multiple settings and by including items from different exams.

## Conclusions

The psychometric properties of the medical qualification exam were inadequate for making valid decisions. However, the difficulty index, efficiency of distracters and item reliability were encouraging. Five option MCQs were not better than four options in terms of psychometric qualities. The qualification examination had a positive predictive validity of future performance. We recommend further capacity-building and continuous mentoring support to improve the item writing skills of instructors. We suggest DTU to assess public health and minor clinical competence independently to ensure the mastery of competence. In addition, DTU should develop a standardized item writing guide and thoroughly evaluate the performance of high-stake exam items before being administered. Furthermore, the findings of the study imply even carefully developed licensing exam items should be subjected to adequate review before administration. We expect the findings of this study will inspire educators to be curious about their assessment tools.

## Supplementary Information

**Additional file 1. **

**Additional file 2. **

## Data Availability

All relevant data analyzed are available in the additional file.
